# Aortic Wall Thickness as a Surrogate for Subclinical Atherosclerosis in Familial and Nonfamilial Hypercholesterolemia: Quantitative 3D Magnetic Resonance Imaging Study and Interrelations with Computed Tomography Calcium Scores, and Carotid Ultrasonography

**DOI:** 10.3390/jcm12175589

**Published:** 2023-08-27

**Authors:** Rafał Gałąska, Dorota Kulawiak-Gałąska, Karolina Dorniak, Aneta Stróżyk, Agnieszka Sabisz, Magdalena Chmara, Bartosz Wasąg, Agnieszka Mickiewicz, Andrzej Rynkiewicz, Marcin Fijałkowski, Marcin Gruchała

**Affiliations:** 1 1st Department of Cardiology, Medical University of Gdansk, 80-210 Gdansk, Polandamickiewicz@gumed.edu.pl (A.M.); mfijalkowski@gumed.edu.pl (M.F.);; 2 Department of Radiology, Medical University of Gdansk, 80-210 Gdansk, Polandagnieszka.sabisz@gumed.edu.pl (A.S.); 3 Department of Noninvasive Cardiac Diagnostics, Medical University of Gdansk, 80-210 Gdansk, Poland; 4 Department of Biology and Genetics, Medical University of Gdansk, 80-210 Gdansk, Polandbwasag@gumed.edu.pl (B.W.); 5 Department of Cardiology and Internal Medicine, University of Warmia and Mazury, 10-727 Olsztyn, Poland; andrzej.rynkiewicz@me.com

**Keywords:** atherosclerosis, familial hypercholesterolemia, cardiovascular magnetic resonance imaging

## Abstract

We aimed to compare the extent of subclinical atherosclerosis in the ascending and descending aortas by measuring wall area and thickness using 3D cardiovascular magnetic resonance imaging (aAWAI and dAWAI) in patients with asymptomatic familial hypercholesterolemia (FH) and nonfamilial hypercholesterolemia (NFH). We also aimed to establish the interrelations of CMR parameters with other subclinical atherosclerosis measurements, such as calcium scores, obtained using computed tomography in coronary arteries (CCS) and ascending and descending aorta (TCSasc and TCSdsc), as well as the carotid intima-media thicknesses (cIMT) using ultrasonography. A total of 60 patients with FH (29 men and 31 women), with a mean age of 52.3 ± 9.6 years, were analyzed. A subclinical atherosclerosis assessment was also performed on a group consisting of 30 age- and gender-matched patients with NFH, with a mean age of 52.5 ± 7.9 years. We found the ascending and descending aortic wall areas and thicknesses in the FH group to be significantly increased than those of the NFH group. A multivariate logistic regression analysis showed that a positive FH mutation value was a strong predictor of high aAWAI and dAWAI independent of the LDL cholesterol level. Correlations across CMR atherosclerotic parameters, calcium scores, and cIMT in the FH and NFH groups, were significant but low. Most of the atherosclerosis tests with high results belonged to the FH group. We found that patients with documented heterozygous FH had a higher atherosclerosis burden in the aorta compared to patients with severe hypercholesterolemia without FH gene mutation. Atherosclerosis is not severe in asymptomatic patients with FH, but is more pronounced and also more diffuse than in patients with NFH. The etiology of hypercholesterolemia, and not just cholesterol levels, plays a significant role in determining the degree of subclinical atherosclerosis.

## 1. Introduction

Familial hypercholesterolemia (FH) is a monogenic, autosomal dominant disease characterized by an elevated low-density lipoprotein cholesterol (LDL-C) level [1]. The genetic variants specific for FH cause significant dysfunction of the LDL receptor (LDLR) pathway leading to severe hypercholesterolemia. It is uncertain whether the FH mutation status provides additional risk information beyond that of the LDL-C measurement [2]. It has been demonstrated that patients who are FH causal variant-positive have a significantly increased cardiovascular risk compared to patients who are FH variant-negative with the same LDL-C levels, and it has also been indicated that across various ranges of observed LDL-C levels, the risk of coronary artery disease (CAD) is elevated in FH mutation carriers in comparison to noncarriers [3]. Within the subgroup of participants with a LDL cholesterol in the 190 to 220 mg/dL range, those with a mutation had a 17-fold increase in CAD risk versus a 5-fold for those without a mutation compared with the reference level. Such striking differences in CAD prevalence suggest that some other mechanisms, possibly related not only to LDL-C differences and cumulative LDL-C cholesterol burden but also to genetic variants, could be responsible for the differences in the severity of atherosclerosis. Although the FH phenotype is heterogenous and is influenced not only by genetic but also various environmental factors [4,5,6], many patients with FH do not develop cardiovascular disease even at an old age [7]. Detection of subclinical atherosclerosis in different vascular locations using noninvasive imaging techniques could improve the understanding of the pathomechanism involved in the early stages of the disease. It allows for early detection of the disease, even in asymptomatic individuals, and could improve risk stratification and the clinical management of patients with FH. Several studies have shown that intimal thickening in the arteries precedes more advanced atherosclerotic lesions and that intimal thickening is more likely to be found in regions of the aorta that are prone to developing atherosclerosis [8,9,10]. Cardiovascular magnetic resonance imaging (CMR) has proven to be useful in the evaluation of the atherosclerosis burden in high-risk patients, including patients with FH, and provides highly reproducible measurements of aortic anatomy and atherosclerosis [11,12]. Measurements of aortic wall thickness and area, as well as measurements of carotid intima-media thickness (cIMT) can be used as surrogate markers for early atherosclerosis, whereas the coronary artery calcium score (CCS) and thoracic aorta calcium score (TCS) assessed using computed tomography are established markers of atherosclerosis [13,14,15]. There are only limited data available on the extent of early atherosclerosis in monogenic FH patients when compared to nonfamilial hypercholesterolemia (NFH). There is a scarcity of studies that simultaneously evaluate multiple vascular beds in patients with FH, especially asymptomatic ones. Thus, we aimed to assess the extent of subclinical atherosclerosis in the ascending and descending aortas of patients with asymptomatic familial hypercholesterolemia (FH) and nonfamilial hypercholesterolemia (NFH) using three-dimensional (3D) cardiovascular magnetic resonance imaging. We also aimed to establish interrelations with other subclinical atherosclerosis measurements, including the CCS, TCS, and cIMT.

## 2. Materials and Methods

### 2.1. Study Population

The study group was selected from patients who had a clinical suspicion of FH and were admitted to our outpatient preventive clinic between 2010 and 2013. For all 156 patients having at least 3 points according to the Dutch Lipid Clinic Network, genomic DNA was isolated from their whole blood using standard methods, and genetic analysis of FH was performed as previously described [16]_._ The inclusion criteria to enter the study included age of above 30 years, at least 3 points according to the Dutch Lipid Clinic Network, and DNA-based diagnosis of FH. The exclusion criteria included previous history of clinically apparent cardiovascular disease and diabetes, secondary hypercholesterolemia due to thyroid or liver disease, renal insufficiency (estimated creatinine clearance <50 mL/min), elevated triglycerides (TG) level > 400 mg/dL, and pregnancy. From the group of included subjects with a DNA-based diagnosis of FH, the group of 60 patients (29 men and 31 women, with a mean age of 52.31 ± 9.62 years) were randomly selected for aortic CMR examination. CMR assessment was also performed on a group of 30 randomly selected individuals matched by age and gender who were diagnosed with severe hypercholesterolemia with an LDL-C level above 190 mg/dL during the same period with nonconfirmed FH causing mutation [1]. The remaining inclusion and exclusion criteria were the same as for the study group. All participants also underwent an ECG-gated cardiac computed tomography and ultrasonography of carotid arteries for cIMT measurement. The Local Ethics Committee approved the study protocol, and written informed consent was obtained from all patients with FH and NFH before enrollment in the study.

### 2.2. Cardiovascular Magnetic Resonance

**Figure 1:**
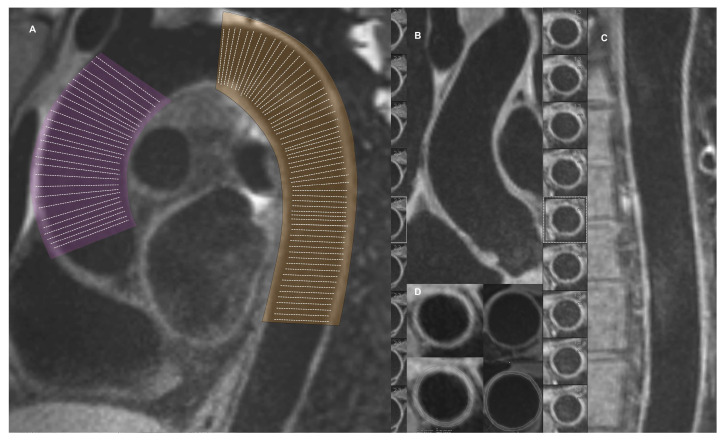
(**A**) An oblique sagittal ‘candy cane’ view of the thoracic aorta with marked cross-sections perpendicular to the vessel centerline that were used for aortic wall area measurement cross-sections. Example of ascending (**B**) and descending (**C**) aorta multiplanar reconstructed short- and long-axis cross-sections using a 3D turbo spin-echo black-blood sequence with a fat saturation impulse and (**D**) an example of short-axis cross-section with manually depicted wall delineation contours.

Participants underwent thoracic aorta CMR using a 1.5-T CMR system (Magnetom Aera, Siemens AG, Erlangen, Germany) with an 18-element phased-array cardiac coil. Scout imaging and transverse, coronal, and oblique sagittal image stacks using a steady-state free precession sequence were first obtained to delineate the anatomy. Then, an oblique sagittal image slab covering the entire thoracic aorta (to enable the so-called ‘candy cane view’) was obtained to achieve an isotropic spatial resolution of approximately 1.3 mm using a T1-weighted 3D turbo spin-echo black-blood sequence with a fat saturation impulse (typical acquisition parameters: acquisition matrix = 320 × 240, flip angle [FA] = 120°, repetition time [TR] = 600 ms, echo time [TE] = 21 ms, number of slices = 60, and slice thickness = 1.3 mm). Image analysis was performed by a cardiologist on the Syngo.via workstation (Siemens). The MR images were visually assessed for overall image quality and aortic wall delineation. Multiplanar curved reconstruction images were prepared, and then cross-sectional images perpendicularly oriented to the long axis of the aorta were generated every 2.5 mm, starting after the origin of the left subclavian artery and stopping at the diaphragm level. Images were magnified so that the transverse dimension of the aorta occupied at least 15 cm on the screen, and the image contrast was indexed. Then, the inner border and the outer border of the aortic wall in each cross-sectional image were manually delineated (Figure 1). The mean aortic wall area (AWA) was calculated for the ascending (aAWA) and descending aorta (dAWA) for each patient using the equation below.

$$AWA=\frac{1}{n}\underset{\begin{array}{c} slices \end{array}}{\sum }WA\;$$

Thus, AWA is a mean aortic area calculated as a sum of areas of all delineated cross-sections, divided by the number of cross-sections. The aortic wall thickness was calculated by dividing the aortic wall area by the average of the inner and outer contour lengths. The indexed wall area was calculated by dividing the aortic wall area by body surface area (BSA). Mean values were computed over all sets, including defined aortic wall area, indexed wall area (aAWAI, dAWAI), and aortic wall thickness. In order to estimate the repeatability of measurements, interobserver and intraobserver variability analysis defined by intraclass coefficient was also performed on 10 patients from both groups for AWA variable, with measurements taken independently by an experienced cardiologist and radiologist. There was a high intra- and interobserver agreement for analyzed AWA variable (interclass correlation coefficient 0.88 and 0.85, respectively, *p* < 0.01).

### 2.3. Computed Tomography

Computed tomography was performed from the aortic arch to the diaphragm during inspiration using a 64-row CT scanner (LightSpeed, GE HealthCare, Chicago, IL, USA). A prospective electrocardiogram-triggered scan protocol without intravenous contrast enhancement at 70% of the RR interval was used with the following scan parameters: slice thickness was 2.5 mm; tube voltage was 120 kV, and tube current 300 mA. Axial images were reconstructed using 512 *×* 512 matrix with an interval of 2.5 mm. Only calcifications with attenuation values >130 HU were used to obtain calcium score result. The location of the aortic calcifications was verified using multiplanar reformats in the three orthogonal planes. Calcium scores were calculated for each participant using the volumetric method with SmartScore v. 4.0 software (GE HealthCare). Calcium scores were obtained for the following regions: coronary arteries (CCS), ascending aorta (TCSasc), and thoracic part of the descending aorta (TCSdsc) to the level of the diaphragm. Calcium score result was defined as sum of all partial values of aortic or coronary artery calcifications in a patient and expressed in mm^3^ (volume score). Details of the image acquisition and treatment procedures have been previously reported [17].

### 2.4. Carotid Intima Media Thickness

All patients had an ultrasound scan of both common carotid arteries, and IMT measurements were performed. Scans were taken using a GE Vivid E9 ultrasound scanner and 4.5–12 MHz linear probe GE 11L (GE HealthCare, Chicago, IL, USA), and they were digitally recorded alongside the ECG reading. The scanning depth was optimized at 3–5 cm. The semiautomatic measurement was taken along the 1 cm distal wall segment at the peak of the ECG R wave using an EchoPAC Clinical Workstation (GE HealthCare, Chicago, IL, USA) with dedicated software. The measurement was taken where the common carotid artery begins to widen, forming a bulb. The mean IMT for a given segment for the left and right carotid arteries were then computed. All measurements were performed twice and then averaged (cIMT). There was a high intra- and interobserver agreement for analyzed variables (interclass correlation coefficient 0.96 and 0.92, respectively, *p* < 0.001). The detailed procedure relating to image acquisition has been reported previously [18].

### 2.5. Statistical Analysis

Continuous variables are shown as means and standard deviations, and if variables were not normally distributed, median values are also presented. Categorical variables are expressed as numbers and percentages. Data normality was tested using the Kolmogorov–Smirnov test. The datasets were compared using the two-sided *t*-test or Mann–Whitney U-test for nonparametric variables depending on the distribution of the variables. Categorical variables were compared using Fisher’s exact test. The correlations between numerical variables were tested by Pearson’s and Spearman’s correlation coefficients, as appropriate. Multivariate logistic regression analysis was used to evaluate the relationships between the clinical characteristics and the high aortic wall area group (N = 30). Analyses were performed using SPSS software v. 26.0. *p* values < 0.05 were considered statistically significant.

## 3. Results

The clinical characteristics of the 60 and 30 patients with FH and NFH, respectively, are presented in Table 1.

**Figure 2:**
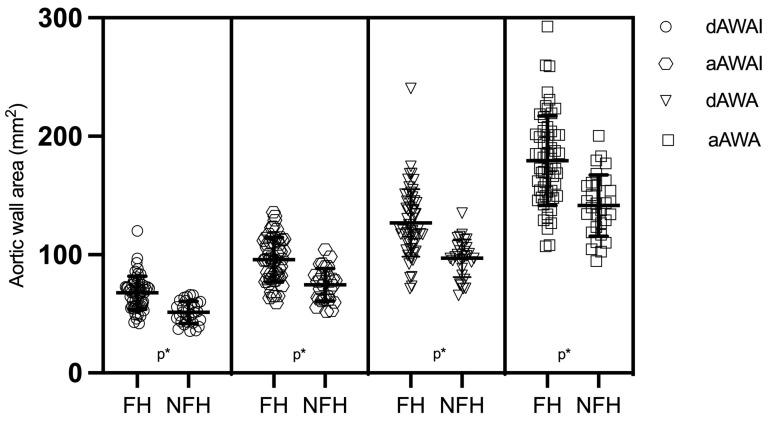
Three-dimensional CMR aortic wall area in the FH and NFH subgroups. Abbreviations: p* < 0.001. FH = familial hypercholesterolemia, NFH = nonfamilial hypercholesterolemia, dAWAI = indexed descending aortic wall area, aAWAI = indexed ascending aortic wall area, dAWA = aortic wall area descending aorta, and aAWA = aortic wall area ascending aorta.

We found no statistical difference in age, body surface area (BSA), systolic blood pressure (SBP), or triglyceride (TG) levels between the FH and NFH groups. The FH group, however, had higher maximum total cholesterol (TCmax), LDL-C max levels, and cholesterol-year scores (TCyear). They also had lower on-treatment high-density lipoprotein cholesterol (HDL-C) levels. The results of the ascending and descending aorta CMR measurements are presented in Table S1 and Figure 2.

We found that the ascending and descending aortic wall areas and thicknesses were significantly higher in the FH group than in the NFH group. This was also true after adjustments were made for BSA. Among the traditional risk factors for atherosclerosis, we observed a strong positive correlation between the CMR parameters and age as well as TCyear and a moderate positive correlation between the CMR parameters and TC-max as well as the LDL-max levels. We also observed significant correlations across subclinical atherosclerosis imaging measurements, including the IMT of both carotid arteries, TCSasc, and CCS (Table S2). Interestingly, we did not observe significant correlations between the CMR parameters and TCSdsc. There were significant and high cross-correlations between aortic ascending and descending CMR parameters (aAWA vs. dAWA, r = 0.77, *p* < 0.01; aAWAI vs. dAWAI, r = 0.84, *p* < 0.01). We sought to determine whether the FH mutation was connected to higher CMR atherosclerosis parameters. For this analysis, the atherosclerosis measurements, aAWAI and dAWAI, in the top tercile of their distribution were categorized as a high level of atherosclerosis. As a result of multivariate logistic regression analysis (Table 2), a positive value of FH mutation was a strong, independent predictor of high aAWAI and dAWAI (OR 16.55, 95% CI 3.44–83.5, *p* = 0.001; OR 17.9, 95% CI 3.63–88.2, *p* = 0.001, respectively). We systematically analyzed other possible confounding variables shown in Table 1 during analysis of our results. The multivariate analysis considered those independent variables that were correlated with aortic wall area but not with each other (age, LDL max, and FH mutation). Among the traditional risk factors, only age was an independent factor for high aAWAI and dAWAI.

**Figure 3:**
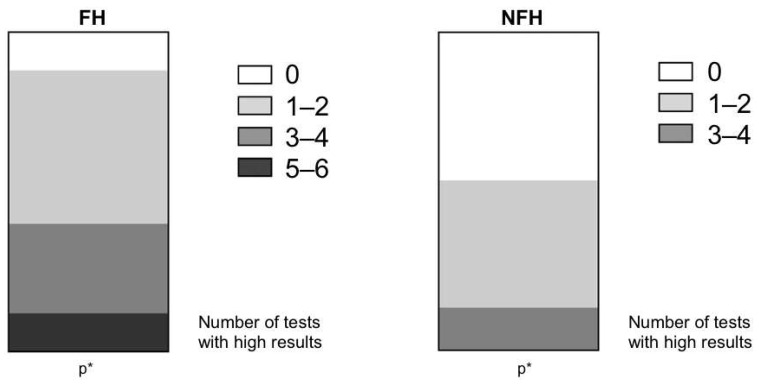
Percentage of FH and NFH patients, with a high atherosclerosis result from 0, 1–2, 3–4, and 5–6 atherosclerosis tests (dAWAI, aAWAI, TCSdsc, TCSasc, CCS, and cIMT). Abbreviations: FH = familial hypercholesterolemia, NFH = nonfamilial hypercholesterolemia, p*—the percentage of patients with at least one high result FH vs. NFH (*p* < 0.001), patients with high results in 5 or 6 tests FH vs. NFH (*p* = 0.05).

Similarly, calcium scores and cIMT measurements in the top tercile were categorized as a high level of atherosclerosis. We separately categorized the FH and NFH groups based on the presence of the FH mutation for each of the six imaging tests: aAWAI dAWAI TCSasc, TCSdsc, CCS, and cIMT. The summarized results for all tests are presented in Table 3. We assessed the number of tests with high results for each subject in both groups (FH and NFH). The results are presented in Table 4. Among atherosclerosis tests based on wall area or IMT thickness (dAWAI, aAWAI, and cIMT), 88.9% of the high results belonged to the FH group. This was especially true for the CMR of the aortic wall area (96.7% and 93.3%, respectively) and 76.7% for cIMT. The majority of high-result tests based on calcium scores (TCSasc, TCSdsc, and CCSvol) were also observed in the FH group, particularly for the ascending aorta (83.3%, 65.2%, and 73.3%, respectively). We also calculated the number of tests with high results for each subject (from 0 to 6, see Figure 3). The percentage of patients with at least one high result was 53.3% in the NFH group and 88.3% (*p* < 0.001) in the FH group. All patients with high results in 5 or 6 tests belonged to the FH group (*p* = 0.05). In a comparison of the high atherosclerosis overlap across aortic CMR, calcium scores, and cIMT, 33.3% of patients with FH and 13.3% of patients with NFH had high atherosclerosis parameters in at least three different vascular beds among ascending aorta, descending aorta, coronary arteries, and carotid arteries (*p* = 0.04).

## 4. Discussion

FH is characterized by a close relationship between genetic variants, severe hypercholesterolemia, and clinical syndromes. In our study, we assessed the extent of subclinical atherosclerosis in the ascending and descending aortas using 3D CMR in asymptomatic patients with severe hypercholesterolemia and a pathogenic FH variant in comparison to patients with severe hypercholesterolemia and without a genetic diagnosis of FH. So far, only a few studies have systematically assessed the severity of atherosclerosis in patients with FH, but to the best of our knowledge, no study has simultaneously compared CMR, CT calcium scores, and cIMT. Our principal finding was that patients with documented heterozygous FH have a significantly higher atherosclerosis burden in the aorta compared to patients with severe hypercholesterolemia without the FH gene mutation. Multivariate analysis showed that the genetic diagnosis of FH, and not the pretreatment LDL-C level, was an independent risk factor for the presence of more advanced atherosclerosis. Consequently, we can conclude that the etiology of elevated cholesterol has a significant impact on the degree of atherosclerotic changes. The significant differences in preclinical atherosclerosis observed could at least partially be explained by patients with FH being exposed to higher LDL-C concentrations since birth than patients with NFH. In our study, the pretreatment LDL-C levels were significantly higher in patients with FH, but because some patients were receiving statin treatment, the total cholesterol year score, which reflects the lifetime cumulative total cholesterol, was only slightly (and significantly borderline) higher in the FH group. Furthermore, it is also possible that the maximum cholesterol level rather than the total cholesterol burden plays an important role in the development of atherosclerotic changes in the aorta [19].

Increased atherosclerosis in the aorta in patients with FH, as assessed by CMR in comparison to healthy controls, has already been well documented. Increased wall thickness of the ascending aorta was demonstrated in nine patients with homozygous familial hypercholesterolemia by Summers et al. [20]. Schmitz et al. studied 11 heterozygous, nonsmoking, nondiabetic, and nonhypertensive patients with heterozygous familial hypercholesterolemia. The patients had a mean age of 44 ± 10 years, and they had been receiving cholesterol-lowering management for a mean of 12 ± 5 years, including 8.25 ± 4.24 years with the highest tolerable doses of a statin or a statin plus ezetimibe, as well as 26 age- and sex-matched control subjects who underwent 3T magnetic resonance imaging of the descending thoracic aorta [11]. These researchers showed that patients with heterozygous familial hypercholesterolemia have a higher aortic wall area and aortic wall thickness than control subjects in quantitative CMR imaging, despite long-term lipid-lowering therapy [21]. Caballero at al. found a higher prevalence of atherosclerotic plaques using CMR in 94% of 36 middle-aged patients with FH (45.7 ± 10.9 years) receiving lipid-lowering treatment, who also showed no evidence of cardiovascular disease, as opposed to 36.8% in the control group of 19 persons without severe hypercholesterolemia [22]. For this study, the researchers selected patients with FH from a group of 811 patients with a clinical diagnosis of FH, which corresponded to a score of ≥6 using an adapted version of the Dutch scoring system. Moreover, 46.4% of this group were current or former smokers, and 30.8% had a null LDLR gene mutation. Both numbers were significantly higher when compared to our group. A null LDLR gene mutation proved to be the strongest risk factor for premature cardiovascular disease, despite the LDL-C levels of the null LDLR gene mutation group not differing significantly from those of the LDLR-defective or -unknown mutation groups.

Based on previous evidence, individuals who are carriers of pathogenic FH variants appear to be at a higher cardiovascular risk when compared to FH variant-negative people, even if they have comparable LDL-C levels. There is a lack of comparative studies on the severity of atherosclerosis in different vascular regions in patients with FH and NFH. In one of the few studies, Sharifi et al. reported that patients with monogenic hypercholesterolemia exhibited a higher severity of carotid and coronary preclinical atherosclerosis than those with a polygenic etiology [23]. There was no significant difference between the monogenic and polygenic groups in pretreatment cholesterol levels. Moreover, the monogenic subjects were younger and had been treated with statins for longer than the polygenic patients. Interestingly, cIMT has been shown to be thicker in patients with monogenic FH, although there was no significant difference in the percentage of patients with carotid plaques between the groups. We compared patients with severe hypercholesterolemia and a genetic diagnosis of FH with patients with hypercholesterolemia who did not have the FH gene mutation. All of them were selected from patients who had at least three points according to the Dutch Lipid Clinic Network, which corresponded to a possible clinical FH diagnosis. There were no significant differences in cholesterol levels at baseline between the groups, but we observed borderline differences in the TC year score value. Nevertheless, we also observed a distinctly higher aortic atherosclerotic burden in the FH group. These changes were observed both in the descending aorta, which is an example of an atherosclerosis-prone artery, and in the ascending aorta, which is an example of an atherosclerosis-resistant artery [8,24]. Moreover, in the subgroup with high atherosclerosis results as assessed by CMR, almost all high measurements were observed in patients with FH. Similarly, almost all high measurements in the ascending aorta calcium scores (TCSasc) were observed in patients with FH, whereas for IMT and calcium scores for CCS, and particularly for TCSdsc, a significant number of patients with NHF were observed to have high atherosclerosis results. These results indicate, as have the results of several previous studies, that atherosclerosis is not distributed equally in the vascular system and might have different susceptibilities to risk factors depending on the mechanism of hypercholesterolemia [25,26,27]. In an interesting study, Kathiresan et al. assessed the thoracic and abdominal aortic plaque burdens using CMR, as well as CCS and TCS using electron beam computed tomography. They assessed common carotid intima-media thickness (cIMT) using ultrasonography in a random sample of 292 participants (mean age 59.5 years, 50% women) from the offspring cohort of the Framingham Heart Study who were free of clinically apparent cardiovascular disease. They found that different participants were identified to have a high atherosclerosis burden by each modality. In a comparison of the overlap across CMR aortic atherosclerosis, calcium scores, and cIMT, only 4% of men and 16% of women from this group were classified as highly atherosclerotic across all three measurements [28]. In our study, we found a higher overlap, particularly in the FH group: 33.3% of patients with FH and 13.3% of patients with NFH had high atherosclerosis parameters in at least three different vascular beds among ascending aorta, descending aorta, coronary arteries, and carotid arteries. Moreover, 30% of patients with FH and 10% of patients with NFH had high atherosclerosis across four or more measurements. These results confirm that atherosclerosis develops in various vascular beds in a substantial proportion of patients with FH and is more advanced than in patients with NFH. Due to the high phenotype variability in patients with FH, additional imaging tests may be useful in risk stratification, e.g., a novel imaging biomarker—the perivascular fat attenuation index—because traditional cardiovascular risk equations are imprecise for FH [29,30]. At present, patients with FH have indications for intensive lipid-lowering treatments, which are usually provided irrespective of such assessments. The presence of atherosclerosis in imaging may be an indication that even more intensive treatment is needed and may be a motivating factor for both the patient and the physician in continuing this treatment. It is especially important for younger asymptomatic patients to avoid other cardiovascular risk factors, such as smoking, obesity, hypertension, and diabetes, which usually increase with age [30,31].

### Limitations of the Study

The principal limitation of this study was the relatively small sample size. However, we observed similar results with two other measurements (carotid IMT and calcium scores) of atherosclerosis burden in previously published papers. Limitations regarding patient selection and FH diagnosis have also been discussed [19,20]. The fact that pretreatment levels of TC max and LDL-C max are significantly different between the FH and NFH groups leads to a potential bias, but we observed only borderline differences in the TC year score values, and there were no significant differences in cholesterol levels at baseline between the groups. Moreover, multivariate logistic regression analysis showed that FH mutation beyond cholesterol levels was an independent risk factor of higher results of atherosclerosis parameters. The actual limitations of noninvasive imaging of coronary atherosclerosis by MRI are mainly technical limitations associated with MRI of the thoracic aorta. The imaging protocol should include sufficient spatial resolution and should allow for the elimination of artifacts due to respiratory motion and pulsatile changes due to blood flow. There are also methodological limitations derived from the lack of generally accepted protocols with standardized sequences for the atherosclerosis assessment of the aorta. In addition, validated automated operator-independent software for quantitative assessment of plaque dimensions and composition is still lacking. The calcium score has been less studied in FH patients as a marker of cardiovascular risk. In our study of asymptomatic patients with FH, we found a significant number of zero calcium scores (45% of CCS, 48% of TCSasc, and 75% of TCSdsc). We found no similar studies on TCS in FH patients, but the CCS results are comparable to percentages reported by other authors [32]. However, interpreting TCSdsc results requires caution, as they may not adequately reflect the difference in the severity of atherosclerotic burden between both groups, owing to the high percentage of zero calcium scores.

## 5. Conclusions

Patients with documented heterozygous FH without previous cardiovascular events had a higher atherosclerosis burden in the aorta, as assessed by magnetic resonance, compared with patients with severe hypercholesterolemia without the FH gene mutation. The etiology of hypercholesterolemia in these patients played a significant role in determining the degree of subclinical atherosclerosis. Atherosclerosis is not severe in asymptomatic patients with FH but is more pronounced and also more diffuse than in patients with NFH. Correlations across atherosclerotic measurements, including CMR aortic wall area and thickness, aortic and coronary calcium scores, and carotid intima-media thickness in the FH and NFH groups were significant but low.

## Figures and Tables

**Table 1:** Clinical characteristics of the FH and NFH groups.

	FH n = 60	NFH n = 30	*p* Value
Age (years)	52.3 ± 9.60	52.5 ± 7.90	ns
Gender	29M 31F	14M 16F	ns
BSA (m^2^)	1.92 ± 0.21	1.89 ± 0.22	ns
TC max (mgL/dL)	353 ± 71.4	313 ± 29.7	<0.01
LDL-C max ^§^ (mgL/dL)	266 ± 63.4	223 ± 26.8	<0.001
HDL-C max ^§^ (mgL/dL)	59.7 ± 15.1	62.9 ± 13.3	ns
TG max ^§^ (mgL/dL)	130 ± 73.8	119 ± 47.1	ns
Chol-year score (mg/dL/year)	17,660 ± 4811	16,620 ± 3666	0.04
TC (mgL/dL)	280 ± 55.11	282 ± 55.1	ns
LDL-C (mgL/dL)	202 ± 73.6	197 ± 48.2	ns
HDL-C (mgL/dL)	56.9 ± 14.3	58.4 ± 11.3	ns
TG (mgL/dL)	118 ± 48.6	122 ± 44.9	ns
SBP (mmHg)	137 ± 16.6	133 ± 15.3	ns
DBP (mmHg)	88.0 ± 9.70	83.2 ± 9.80	0.03
Smoking ^¶^ (n)	20 (33%)	16 (53%)	ns
Statin treatment on 1st visit (n)	33 (58%)	12 (50%)	ns

^§^ = data not available in 5 FH patients, ^¶^ smoking = never > 1 pack/year. Abbreviations: FH = familial hypercholesterolemia, NFH = nonfamilial hypercholesterolemia, ns = not significant, BSA = body surface area, TC = total cholesterol, LDL-C = low-density lipoprotein cholesterol, HDL-C = high-density lipoprotein cholesterol, TG = triglycerides, max = maximum levels without pharmacotherapy, SBP = systolic blood pressure, DBP = diastolic blood pressure.

**Table 2:** Multivariate logistic regression analysis for identification of patients with high aAWAI and dAWAI (N = 90, high aAWAI N = 30, and high dAWAI N = 30).

N = 90	aAWAI Odds Ratio (±95% CI)	*p* Value	dAWAI Odds Ratio (±95% CI)	*p* Value
Age	1.08 (1.02–1.15)	0.006	1.08 (1.02–1.15)	0.009
FH	16.6 (3.44–83.5)	0.001	17.9 (3.63–88.2)	0.001

Abbreviations: aAWAI = indexed ascending aortic wall area, dAWAI = indexed descending aortic wall area, and FH = FH receptor mutation.

**Table 3:** Subclinical atherosclerosis imaging test values.

		FH	NFH	*p* Value
aAWAI (mm^2^)	mean ± SD T2	95.7 ± 19.7	74.7 ± 13.5	0.001
		97.6		
dAWAI (mm^2^)	mean ± SD T2	67.8 ± 14.1	51.9 ± 9.8	0.001
		68.2		
cIMT (mm)	mean ± SD T2	0.73 ± 0.19	0.66 ± 0.12	0.03
		0.74		
CCS (mm^3^)	median mean ± SD T2	5.5 60.5 ± 166.2	3 32.9 ± 87.8	ns
		20		
TCSasc (mm^3^)	median mean ± SD T2	2 32.3 ± 61.9	0 4.8 ± 10.6	0.005
		15		
TCSdsc (mm^3^)	median mean ± SD T2	0 17.7 ± 58.3	0 9.5 ± 19.8	ns
		NA		

Values are expressed as mean ± SD and median values for not normally distributed results. T2—high atherosclerosis result (top tercile). Abbreviations: FH = familial hypercholesterolemia, NFH = nonfamilial hypercholesterolemia, aAWAI = indexed ascending aorta wall area, dAWAI = indexed descending aorta wall area, TCSasc = ascending aorta thoracic calcium score, TCSdsc = descending aorta thoracic calcium score, CCS = coronary calcium score, cIMT = intima-media thickness, and NA—not applicable (23 nonzero TCS results were observed in both groups, and the value of T2 = 0 for TCSdsc).

**Table 4:** Proportion of high atherosclerosis results in FH and NFH subgroups across different atherosclerosis tests.

Proportion of High Atherosclerosis Results (%)	FH (n = 60)	NFH (n = 30)	*p* Value
dAWAI	48.3 (29/60)	6.7 (1/30)	<0.001
aAWAI	46.7 (28/60)	6.7 (2/30)	<0.001
cIMT	38.0 (23/60)	23.3(7/39)	ns
TCSdsc	25.0 (15/60)	26.7 (8/30)	ns
TCSacs	41.7 (25/60)	16.7 (5/30)	<0.001
CCS	36.7 (22/60)	26.7 (8/30)	ns

Abbreviations: FH = familial hypercholesterolemia, NFH = nonfamilial hypercholesterolemia, aAWAI = indexed ascending aorta wall area, dAWAI = indexed descending aorta wall area, TCSasc = ascending aorta thoracic calcium score, TCSdsc = descending aorta thoracic calcium score, CCS = coronary calcium score, and cIMT = intima-media thickness.

## Data Availability

The datasets generated during and/or analyzed during the current study are available from the corresponding author on reasonable request.

## Supplementary Materials

The following supporting information can be downloaded: https://www.mdpi.com/article/10.3390/jcm12175589/s1, Figure S1: Pretreatment cholesterol levels. Table S1: 3D CMR aortic wall thickness and diameter in FH and NFH subgroups. Table S2: Correlations across subclinical atherosclerosis imaging parameters.
