# Supplementary material for: Aortic Wall Thickness as a Surrogate for Subclinical Atherosclerosis in Familial and Nonfamilial Hypercholesterolemia: Quantitative 3D Magnetic Resonance Imaging Study and Interrelations with Computed Tomography Calcium Scores, and Carotid Ultrasonography
Source: J Clin Med. 2023 Aug 27;12(17):5589. doi: 10.3390/jcm12175589 (PMC10488167; doi:10.3390/jcm12175589)
Supplement: Supplementary file 1 [file jcm-12-05589-s001.zip › jcm-2525502-supplementary.pdf]

Table S1. 3D CMR aortic wall thickness and diameter in FH and NFH subgroups.

	**FH**	**NFH**	**p**
dThick (cm)	0.163 ± 0.028	0.128 ± 0.014	<0.001
dD (cm)	2.452 ± 0.254	2.432 ± 0.238	ns
aThick (cm)	0.172 ± 0.029	0.135 ± 0.0132	<0.001
aD (cm)	3.320 ± 0.374	3.318 ± 0.318	ns

Abbreviations:

FH = familial hypercholesterolemia, NFH = nonfamilial hypercholesterolemia, ns = not significant, dD = mean descending aortic diameter, aD = mean ascending aortic diameter, dThick = mean descending aortic wall thickness, and aThick = mean ascending aortic wall thickness.

Table S2. Correlations across subclinical atherosclerosis imaging parameters.

	**aAWA**	**aAWAI**	**dAWA**	**dAWAI**
TCSasc	0.38***	0.37***	0.36***	0.29**
TCSdsc	0.16	0.1	0.21	0.19
CCS	0.33**	0.23*	0.27**	0.17
cIMT	0.46***	0.27*	0.35***	0.3**

p< 0.05, **p< 0.01, *** p< 0.001

Abbreviations:

CMR = cardiovascular magnetic resonance, aAWA = mean ascending aortic wall area, dAWA = mean descending aortic wall area, aAWAI = indexed ascending aortic area, dAWAI= indexed descending aortic area, TCSasc = ascending aorta thoracic calcium score, TCSdsc descending = descending aorta thoracic calcium score, CCS = coronary calcium score, and cIMT = intima media thickness.

Figure S1. Pretreatment cholesterol levels

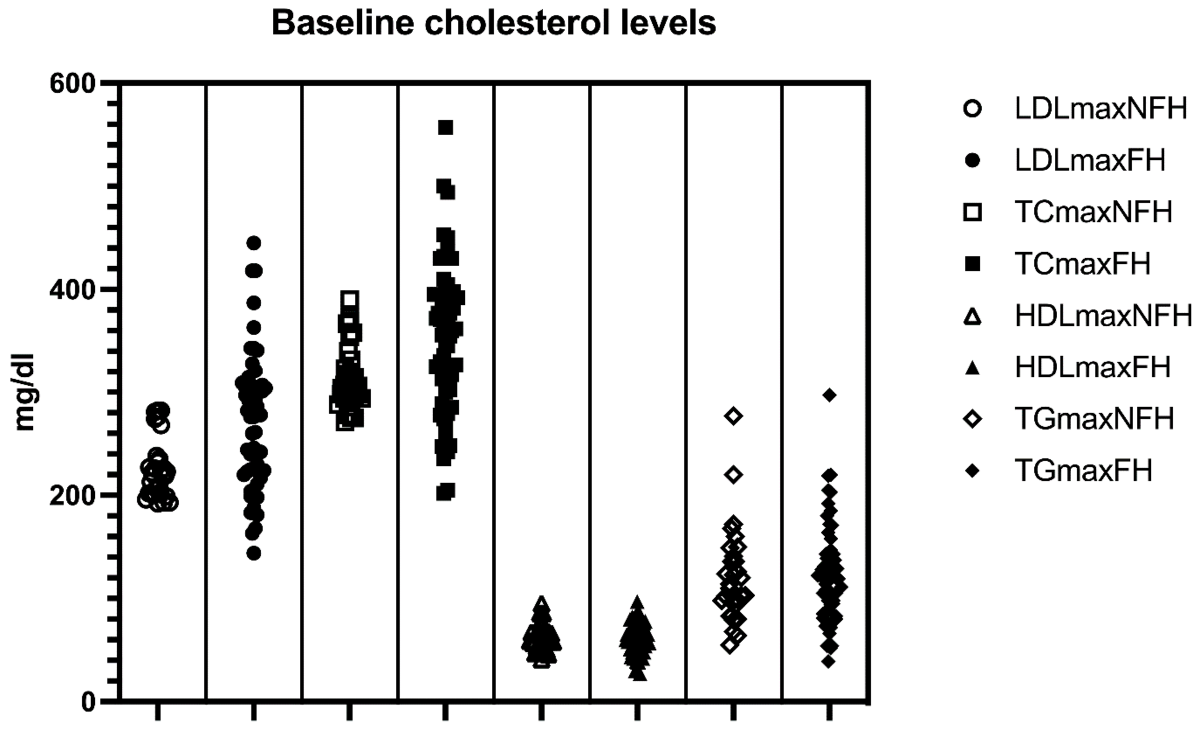

Abbreviations:

FH = familial hypercholesterolemia, NFH = nonfamilial hypercholesterolemia, TC = total cholesterol, LDL-C = low-density lipoprotein cholesterol, HDL-C = high-density lipoprotein cholesterol, TG = triglycerides, max = maximum levels without pharmacotherapy
